# Functional characterization of a *GFAP* variant of uncertain significance in an Alexander disease case within the setting of an individualized medicine clinic

**DOI:** 10.1002/ccr3.655

**Published:** 2016-08-15

**Authors:** Nicole J. Boczek, Ashley N. Sigafoos, Michael T. Zimmermann, Rachel L. Maus, Margot A. Cousin, Patrick R. Blackburn, Raul Urrutia, Karl J. Clark, Marc C. Patterson, Myra J. Wick, Eric W. Klee

**Affiliations:** ^1^ Center for Individualized MedicineMayo ClinicRochesterMinnesotaUSA; ^2^ Department of Biochemistry and Molecular BiologyMayo ClinicRochesterMinnesotaUSA; ^3^ Department of Biomedical InformaticsMayo ClinicRochesterMinnesotaUSA; ^4^ Mayo Graduate School and the Department of ImmunologyMayo ClinicRochesterMinnesotaUSA; ^5^ Department of Biophysics and MedicineMayo ClinicRochesterMinnesotaUSA; ^6^ Department of Clinical GenomicsMayo ClinicRochesterMinnesotaUSA; ^7^ Departments of Neurology and PediatricsMayo ClinicRochesterMinnesotaUSA; ^8^ Department of Obstetrics and GynecologyMayo ClinicRochesterMinnesotaUSA

**Keywords:** Alexander disease, developmental delay disorders, glial fibrillary acid protein, leukoencephalopathies, muscle hypotonia, paralog analysis

## Abstract

A de novo GFAP variant, p.R376W, was identified in a child presenting with hypotonia, developmental delay, and abnormal brain MRI. Following the 2015 ACMG variant classification guidelines and the functional studies showing protein aggregate formation in vitro, p.R376W should be classified as a pathogenic variant, causative for Alexander disease.

## Introduction

Next‐generation sequencing has ushered in an era of individualized medicine enabling a patient's genome to be interrogated in search of variants implicated in disease. This has brought about diagnostic resolution for many patients suffering from previously undiagnosed genetic disorders. However, it has also created significant challenges in clinical interpretation, as many identified variants lack sufficient contextual information to allow clear association with disease and are classified as variants of uncertain significance (VUS). Here, we describe the sequential use of variant annotation, in silico protein modeling techniques, and in vitro functional studies to characterize a clinically reported VUS and reclassify it as a pathogenic variant.

In this study, genetic panel testing in a 19‐month‐old boy with developmental delay, hypotonia, and abnormal brain MRI revealed a VUS, c.1126C>T, leading to p.R376W, in the glial fibrillary acidic protein (GFAP; OMIM:203450; NM_002055.4) which has previously been associated with Alexander disease (OMIM: 203450) 1. Alexander disease, a rare genetic disorder with more than 550 cases reported worldwide, is characterized as a progressive disorder of cerebral white matter 2. Alexander disease can present during infancy, childhood, or adulthood, with infantile onset being the most common presentation. The infantile form often presents with progressive psychomotor retardation, loss of developmental milestones, frontal bossing with megalencephaly, seizures, hyperreflexia, ataxia, and hydrocephalus, and frequently leads to death within the first decade 2. Juvenile and adult forms of Alexander disease often progress more slowly with ataxia, bulbar signs, and spasticity 3.

The predominant genetic cause of Alexander disease was discovered after transgenic *GFAP* mice presented with astrocyte inclusions that pathologically mimicked the Rosenthal fibers in Alexander disease 4. The mouse model led to targeted *GFAP* sequencing in 11 cases of Alexander disease, 10 of which harbored a genetic variant. For cases with available parental samples, the variants were found to have arisen as de novo in the proband, and were absent in their larger cohort of control DNA samples 1. Since these initial studies, over 110 distinct pathogenic variants in GFAP have been identified 2. The vast majority of these variants are de novo missense heterozygous variants; however, there is some evidence supporting familial inheritance 5.

Following the initial discovery of *GFAP* variants associated with Alexander disease, the functional characterization of newly identified variants was completed in mouse models 6. However, the vast majority of subsequently identified missense variants in *GFAP* were considered pathogenic without functional validation. Cellular‐based assays have been developed and used to examine the pathogenicity of variants in individuals with abnormal presentation or familial onset 3, 5. Results of these studies demonstrated that some variants elicit a wild‐type phenotype, suggesting they were benign in nature 3. These cases highlight the need for functional validation of *GFAP* variants associated with atypical Alexander disease.

As our patient presented with developmental delay, hypotonia, and abnormal brain MRI, with no signs of regression, it could not confidently be concluded that the identified *GFAP* variant was associated with his disease phenotype. Because his phenotype may represent a subtle and atypical form of Alexander disease, we combined variant annotation using information on variant frequency in Alexander disease cases and publically available exome databases, protein studies via paralog modeling, and functional characterization utilizing a cellular model to determine the pathogenicity of p.R376W.

## Materials and Methods

### Study subject

The patient was seen at Mayo Clinic and referred to the Center for Individualized Medicine by Medical Genetics for further variant interpretation and functional validation studies. Mayo Clinic's Institutional Review Board does not require consent for single‐patient studies; however, consent to publish was obtained. In addition, genetic testing completed on the patient was performed as part of a clinical genetics evaluation and was approved by the patient's family. All research‐based questions completed in this study did not use any patient materials.

### GFAP mammalian expression vectors

GFAP cDNA was purchased from GE Dharmacon (Lafayette, CO) and subcloned into the pKTol2C‐GFP (green fluorescent protein) plasmid 7. Using primers listed in Table S1, we amplified GFAP cDNA and subcloned the resulting fragments into the pKTol2C‐GFP cut with XhoI and BglII using a Gibson Assembly Cloning Kit (New England Biolabs, Ipswitch, MA). This cloning replaced the GFP coding sequence with GFAP. Full‐length GFAP and variants p.R239H, p.S247P, p.A253G, and p.R376W were all introduced into the pkTol2C‐GFAP construct. The integrity of all constructs was verified through Sanger DNA sequencing.

### SW‐13 cell culture, transfection, and immunofluorescence

In order to examine fiber formation in GFAP, previously established methods were followed 3, 5. In summary, SW‐13 cells (ATCC, Manassas, VA) were cultured in Lebovitz's culture medium (ATCC, Manassas, VA) supplemented with 10% fetal bovine serum, and 1% penicillin/streptomycin/glutamine solution (CellGro, Manassas, VA) in a 0% CO_2_ incubator at 37°C. Cells were split and allowed to grow on 22‐mm glass coverslips for 24 h. Heterologous expression of GFAP was accomplished by cotransfecting 1 *μ*g *GFAP* wild‐type (WT) or variant cDNA with 3 *μ*L Lipofectamine 2000 in OPTI‐MEM media. The media were replaced with fresh Lebovitz's culture medium after 4–6 h. Twenty‐four hours post‐transfection, immunofluorescence experiments began. Cells were rinsed with 4°C phosphate‐buffered saline (PBS) three times and were subsequently fixed using 5% paraformaldehyde in PBS. Cells were washed in PBS and permeabilized using 0.1% Triton X‐100 in PBS. Cells were then washed with PBS with 0.05% Tween‐20 (PBST) and subsequently blocked with 10% normal goat serum (Life Technologies, Grand Island, NY). Cells were incubated in primary antibodies at room temperature, 1:2000 rabbit polyclonal anti‐cow GFAP (DAKO, Carpinteria, CA) and 1:2000 mouse monoclonal anti‐vimentin (Sigma, St. Louis, MO). Subsequently, cells were washed in PBST and blocked in 10% goat serum. The secondary antibodies (1:100), Alexa Fluor 488‐conjugated goat anti‐rabbit IgG and Alexa fluor 594‐conjugated goat anti‐mouse IgG (Molecular Probes, Grand Island, NY), were incubated on cells at room temperature. Cells were washed again in PBST and mounted to slides using Vectashield mounting media with DAPI stain (Vector Laboratories, Burlingame, CA). Vimentin staining was utilized as a negative screen, so we could examine only those cells not expressing vimentin protein. In addition, cells stained with only secondary antibodies were utilized as a negative control. Cells were then imaged using a Zeiss Laser Scanning Microscope 780.

### Protein annotation and modeling

The monomer sequence was annotated according to the presence of known autosomal dominant variants (http://www.waisman.wisc.edu/alexander-disease), variants indexed by the Intermediate Filament Database 8, pathogenic variants from ClinVar 9, and HGMD 10. The domain architecture used was according to Uniprot (P14136) 11. Paralog annotation analysis was performed using a previously described method 12. Briefly, for this purpose multiple sequence alignment (MSA) of paralogs was generated using paralog sequences gathered from Ensembl 13 gene ENSG00000131095 using annotations from GRCh37.p13. The MSA was constructed using Clustal Omega 14, 15 with default parameters. Published variants in paralogs were obtained from PubMed and projected on the sequence of human GFAP. Protein secondary structure was predicted using PsiPred 16. Homology‐based protein structure models were constructed using Modeller 17, 18 version 9.15. The tetramer was generated from homology‐based modeling using a vimentin tetramer model 19, 20 as a template. Intra‐ and intermolecular interactions, including salt bridge interactions, hydrogen bonds, electrostatic interactions, and hydrophobic interactions, were calculated in the Receptor‐Ligand function of Discovery Studio Client 4.0 21. In silico mutagenesis was performed using the Accelrys Builder software 21. Molecular models were first energy minimized using a two‐step protocol of steepest descent and conjugated gradients. All these steps were performed using the SHAKE procedure. A distance‐dependent dielectrics implicit solvent model was used with a dielectric constant of 80 and a pH of 7.4 as previously described 21.

## Results

### Phenotype‐to‐genotype correlation of a leukodystrophy‐affected infant harboring a p.R376W GFAP variant

**Figure 1:**
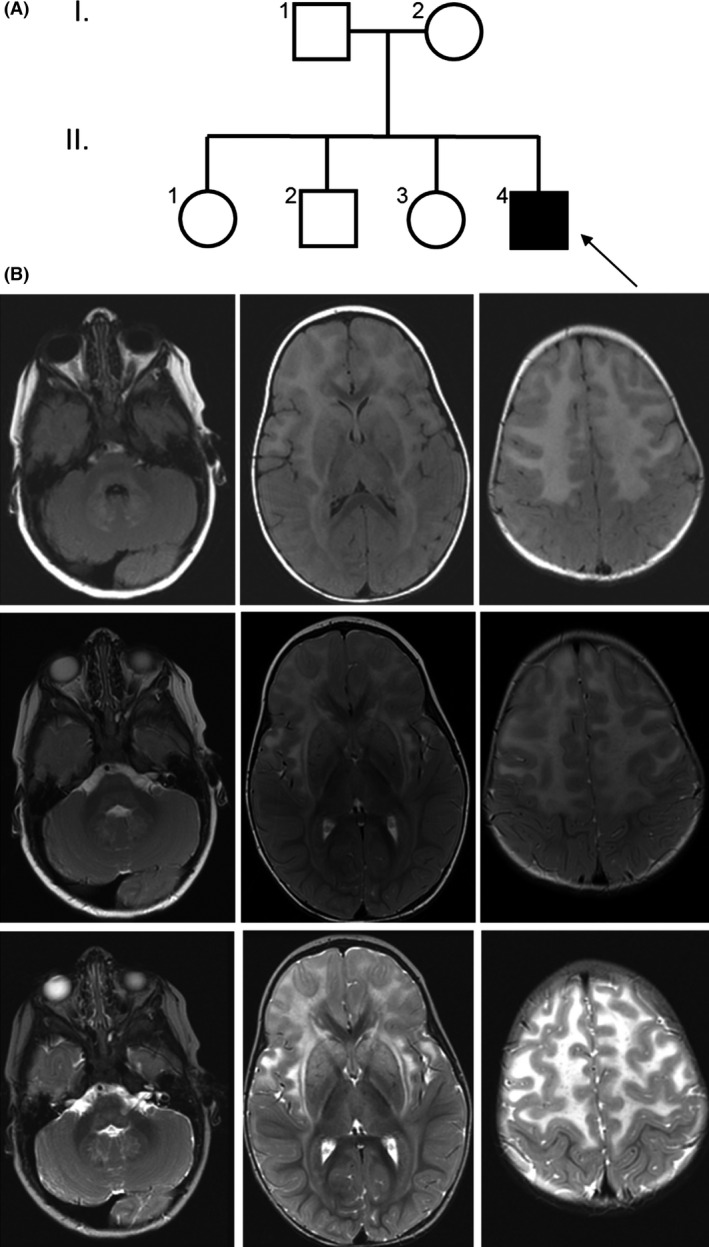
Brain MRI for the Proband. (A) Pedigree of the family with the proband demarcated with an arrow. (B) Brain MRI images of the proband at 18 months of age. Axial MRI images (left to right) at the level of the orbits, foramen of Monro, and supraventricular centrum semiovale, respectively, utilizing (upper to lower row, respectively) T2 FLAIR, axial T2 FSE Pre, and FSE‐IR imaging sequences. The images show signal hyperintensity in the frontal white matter, external capsules, thalami, striatum, middle cerebellar peduncles, dentate nuclei, medulla, and periventricular white matter, typical of Alexander disease.

The proband was the fourth child born to a healthy 32‐year‐old gravida four para four female and 33‐year‐old male, both of European Caucasian descent (Fig. 1A). There was no report of consanguinity and the family histories were noncontributory. The pregnancy was uncomplicated, birth weight was 3.7 kg, Apgar scores were unremarkable, and standard newborn screening was normal with the exception of hypotonia, and difficulties with the mechanics of breastfeeding. There were no concerns regarding early developmental delay. At 6 months of age, he developed protracted vomiting which resulted in poor weight gain; weight gain improved after ranitidine was prescribed at 9 months of age.

Between 6 and 9 months of age, concern for developmental delay prompted a brain MRI which was concerning for leukodystrophy. Electroencephalogram was normal. An extensive diagnostic workup was negative and included the following tests: 7‐dehydrocholesterol, urine organic acids, urine mucopolysaccharidosis screen, urine and serum amino acids, iron, lactic acid, folate, CK, TSH, CMP, EEG, lysosomal studies, microarray, and congenital disorders of glycosylation. Very long‐chain fatty acids (peroxisomal studies) and uric acid levels were also evaluated and were essentially normal. Based on the abnormal brain MRI findings, genetic testing of the proband was completed at Medical Neurogenetics using an 84‐gene leukodystrophy panel. This test identified a de novo missense variant, c.1126C>T, in *GFAP*, which was deemed a variant of uncertain significance (VUS). No other variants were reported.

Based on the association of GFAP variations with Alexander disease and due to the atypical and somewhat subtle presentation of neurological sequelae in our patient, additional phenotypic information was collected to improve our phenotype‐to‐genotype association. Developmental milestones included sitting at 11 months of age. Around 18 months, he was also able to army crawl, pull to stand, sign, and vocalize a few words. He showed impaired fine motor skills, drank through a straw, and could only eat baby food. Hypotonia, hypermobility, and difficulty in weight bearing through both the lower and upper extremities were noted. At 18 months, his height was 80.3 cm (22nd percentile), weight 10.6 kg (37th percentile), and his head circumference 40 cm (89th percentile), which was suggestive of relative macrocephaly. A GI video swallow study showed premature spillage over the base of the tongue and a delayed swallow, but no laryngeal penetration or aspiration.

In addition, a follow‐up brain MRI at 18 months was obtained and showed abnormal T2 white matter signal with a frontal predominance, which involved the white matter surrounding the basal ganglia, the brainstem, and the dentate nuclei. There was patchy enhancement, particularly adjacent to the frontal horns. MR spectroscopy demonstrated reduced N‐acetyl aspartate acid, elevated choline, and probable lactate elevation, which was most pronounced in the right frontal lobe (Fig. 1B). These MRI findings led us to reconsider the candidacy of c.1126C>T as an Alexander disease *GFAP* variant. Therefore, we pursued paralog annotation and functional validation experiments to confirm the pathogenicity of this variant.

### Impaired function of the GFAP p.R376W variant supports its pathogenic role in Alexander disease

The c.1126C>T variant resides in exon 6 of *GFAP*, and is predicted to result in an arginine to tryptophan substitution of amino acid position 376 (p.R376W), in the C‐terminus of GFAP. This amino acid is completely conserved throughout vertebrates, is predicted to be deleterious by in silico prediction tools including SIFT and Polyphen2, and is absent in the Exome Aggregation Consortium (ExAC; *n* = 60,706) 22.

**Figure 2:**
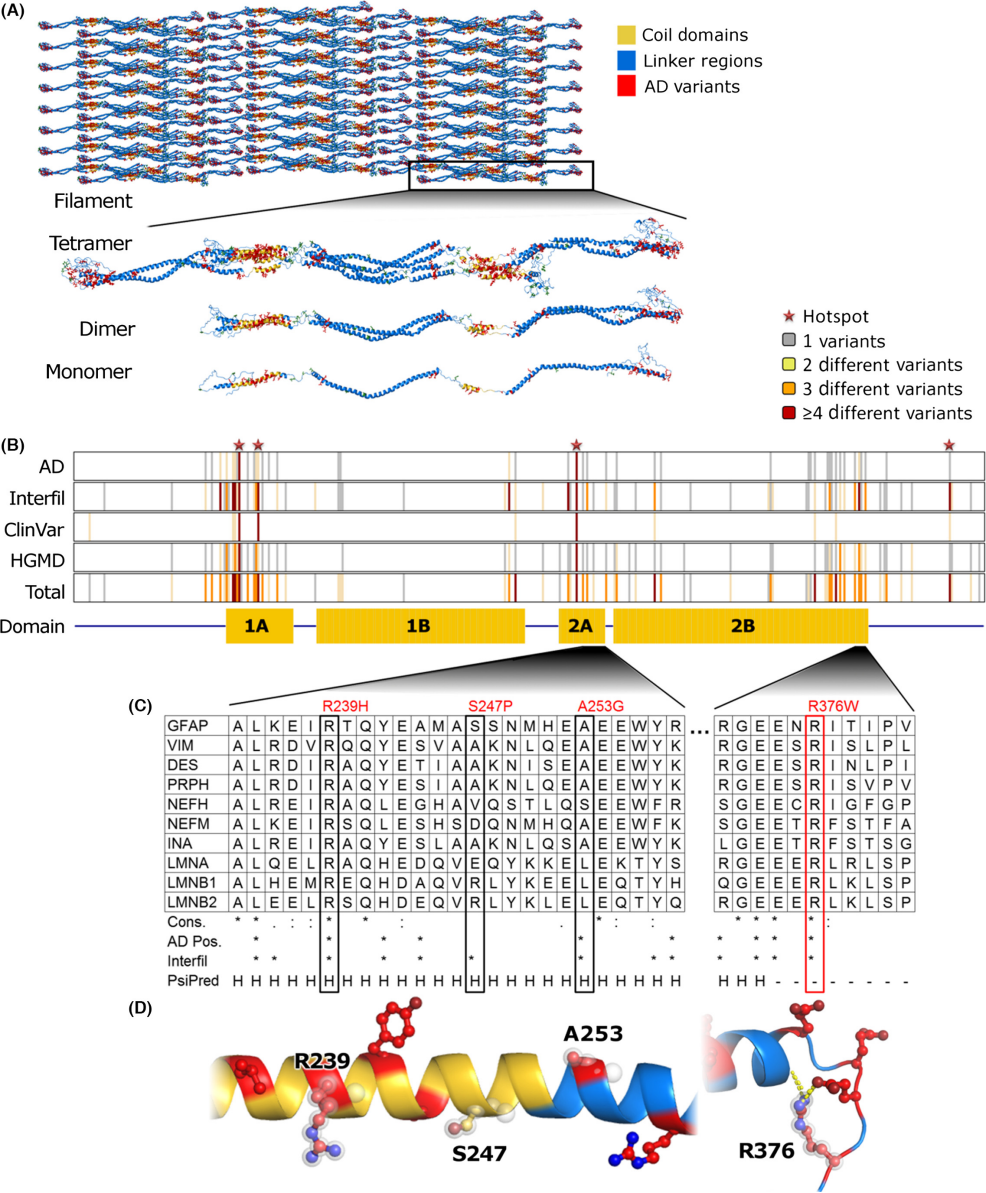
Annotation of Clinically Observed GFAP Variant Reveals Likely Pathologic Effects. (A) Representations at each level of the assembled GFAP filament generated from a model of the GFAP tetramer. Helical regions within the coil domains are colored yellow, positions with known Alexander disease variants red, and other residues blue. (B) We annotated the monomer sequence according to the presence of known variants from multiple databases and the domain architecture. (C) The four variants considered in this study are shown within the context of an MSA of paralogs. Below the MSA, we show sequence conservation (similarity indicated by a semicolon and identity by an asterisk), followed by presence (indicated by an asterisk) of disease‐associated variants in each position of GFAP, and PsiPred prediction of protein secondary structure, with H representing helix formation and – representing a coil. (D) Segments of the protein structure generated by homology modeling are shown, colored as in (A) and with the four resides considered in this study highlighted.

GFAP is an intermediate filament type III (IF III), composed of helical coiled domains and linker regions, and is known to assemble in large filament complexes as observed in Figure 2A. Although the structure of GFAP has not been elucidated, there are IF III paralogs with close sequence homology in which the structure has been previously described. Annotations from well‐characterized pathogenic variants in these proteins inform on the likely physiology for analogous variants in GFAP. To examine these similarities, the paralog annotation method, which has recently been successfully applied to better define channelopathy‐associated variants 12, 23, was utilized. The results of these analyses revealed that p.R376W affects a highly conserved residue which is located within the C‐terminal region of GFAP (Fig. 2B–D). This domain has been shown to be critical for the formation of GFAP intermediate filaments 24. In fact, our paralog annotation analyses show that this region of IF III proteins is a common hotspot for disease‐causing variants. For example, desmin (DES) and lamin A/C (LMNA) are paralogs to GFAP and also assemble into IF IIIs. GFAP R376 corresponds to DES R415. This residue is flanked by residues with pathogenic variants in desmin: p.E413K 25, p.E413R 26, and p.P419S 27, 28, which all led to autosomal dominant myofibrillar myopathies. This C‐terminal region is necessary for fibril formation 29. Within LMNA, the analogous arginine (R274 or R386 for short and long isoforms, respectively) is reported in both the ClinVar and HGMD databases and has been observed in a small number of case studies of muscular dystrophy 30, 31. Thus, this analysis suggested that p.R376W may alter normal GFAP function; therefore, functional studies were performed to test for the validity of this hypothesis.

**Figure 3:**
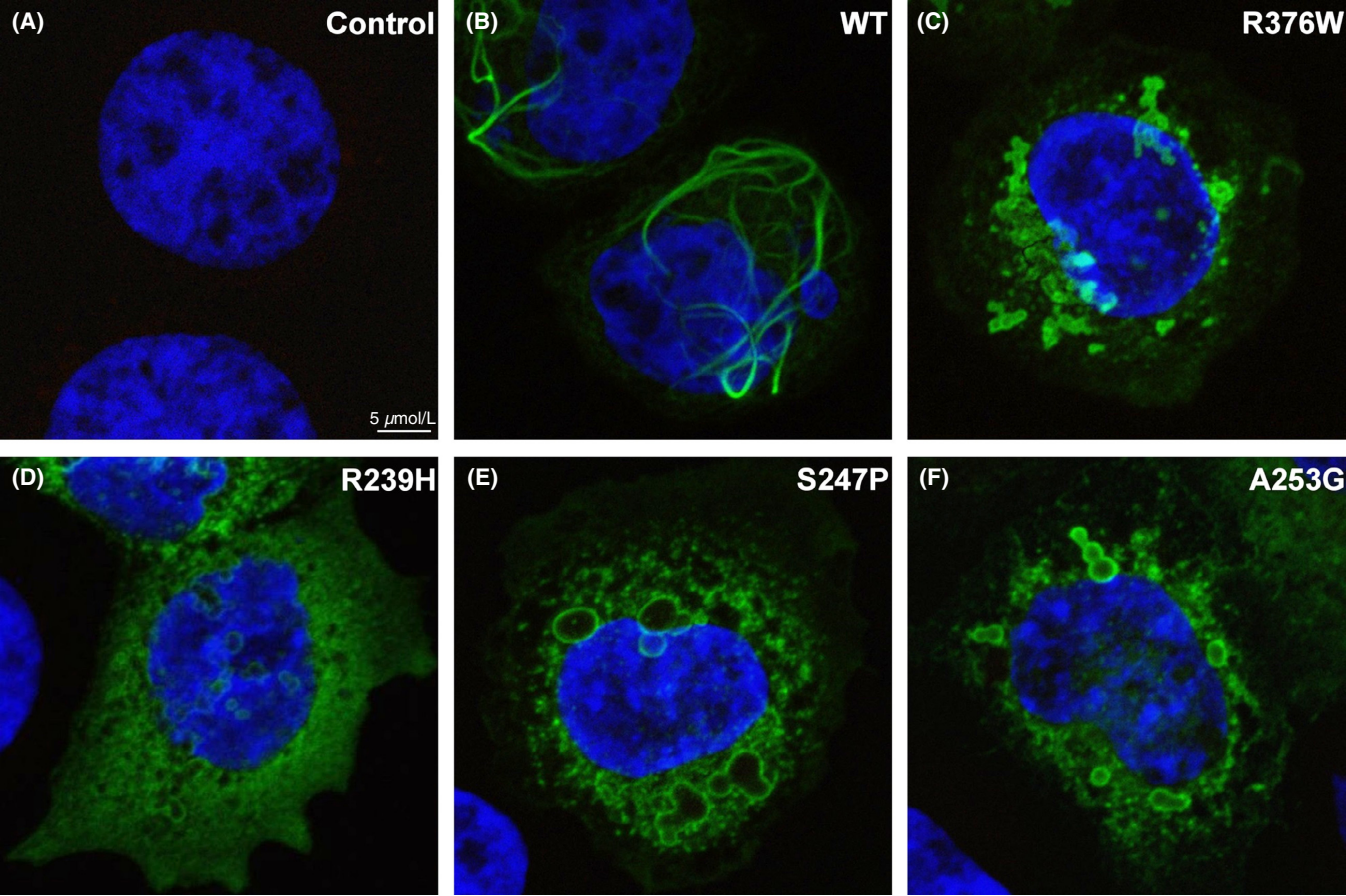
Immunofluorescence of WT and Mutant GFAP. SW‐13 cells transiently transfected with WT or mutant GFAP were stained with vimentin and GFAP antibodies. Fluorescent secondary antibodies labeling GFAP with green fluorescence and vimentin with red fluorescence were utilized to visualize these proteins using confocal microscopy. The mounting media for the cells contained DAPI stain, which was utilized to visualize the nucleus of each cell. First, only secondary antibodies were utilized to ensure there was no cross‐contamination (A). Primary and secondary antibodies were utilized to examine cells expressing GFAP, which were not concurrently expressing vimentin. These experiments were completed and representative cells are shown for WT‐GFAP (B), p.R376W‐GFAP (C), p.R239H‐GFAP (D), p.S247P‐GFAP (E), and p.A253G‐GFAP (F).

A previously described in vitro cell‐based assay was used to assess the impact of GFAP variants on filament formation, which, if aberrant, would suggest variant pathogenicity 3, 5. Wild‐type and p.R376W GFAP were transfected into SW‐13 cells and imaged for the formation of intermediate filaments by immunofluorescence using a laser‐assisted confocal microscope. For easier interpretation of the phenotype, cells negative for vimentin staining were imaged. As positive controls, cells were transfected with cDNA constructs encoding well‐characterized known pathogenic GFAP variants which disrupt filament formation, namely p.R239H, p.S247P, and p.A253G. The p.R239H variant has been previously modeled in mice as pathogenic, 32 but has not however, been characterized using this cellular model. The p.S247P 5 and p.A253G 3 have specifically been modeled using the same cellular assay as utilized in this study. The results of these studies, shown in Figure 3, demonstrate that, in contrast with the wild‐type, but similar to known pathogenic variants (p.R239H, p.S247P, and p.A253G), p.R376W impairs intermediate filament formation, suggesting pathogenicity.

## Discussion

Genetic testing can be useful for identification of rare disorders with nonclassic phenotypes. In this study, we report on a patient with multiple abnormalities including developmental delay and abnormal brain MRI. Based on his abnormal brain MRI, a leukodystrophy gene panel was ordered, revealing a de novo VUS in GFAP, p.R376W. Variants in GFAP are associated with Alexander disease, and although our patient showed several features of Alexander disease, he had an atypical progression of disease with no signs of regression and a less severe presentation of symptoms. Therefore, confirmatory functional testing was necessary to establish the pathogenicity of the detected variant.

Alexander disease has been classified into three major subtypes, infantile, juvenile, and adult onset. Each subtype has general phenotypic sequelae that are associated with disease manifestation. The infantile form is the most common type of Alexander disease with onset during the first 2 years of life, and is characterized by progressive psychomotor delay and regression of developmental milestones, frontal bossing and megalencephaly, seizures, hyperreflexia, ataxia, and hydrocephalus. Individuals presenting during the infantile period often do not survive past adolescence. The juvenile form is the least common type of Alexander disease, with onset between 4 years of age and the teenage years. Symptoms include bulbar signs, lower limb spasticity, poor coordination, intellectual regression, seizures, megalencephaly, and breathing problems. Individuals with the juvenile form have variable survival, with death occurring in the teens to third decade. The adult form of Alexander disease can be highly variable and often has slower progression compared with the other Alexander disease subtypes. The most typical features include bulbar signs, pyramidal tract signs, cerebellar signs, dysautonomia, sleep disturbance, gait abnormality, seizures, and diplopia. Individuals with the adult form can survive several years to many decades after the onset of symptoms 2.

Despite phenotypic variability of Alexander disease, the only genetic substrate that has been associated with Alexander disease to date is *GFAP*. The vast majority of individuals with clinically diagnosed Alexander disease have positive genetic testing most often revealing de novo variants within *GFAP;* however, there have been some examples of familial Alexander disease with appropriate cosegregation. Although it has been widely assumed that most missense variants in *GFAP* in affected individuals contribute to disease, there have been several examples of *GFAP* variants with functional characterization providing evidence that the variant did not lead to abnormal fiber formation, suggesting benign consequences 3.

*GFAP,* located on chromosome 17 (17q21), encodes for the glial fibrillary acid protein. GFAP is an IF III with a tripartite structure, with a non‐*α*‐helical N‐terminal head domain, followed by an *α*‐helical rod domain, followed by a non‐*α*‐helical C‐terminal tail domain (Fig. 2). Intermediate filaments assemble together and form coiled‐coils using the rod domain, while the head and tail domains facilitate interfilament assembly 33. The variant identified in our patient, p.R376W, resides in the intrinsically disordered region of the tail domain. The arginine to tryptophan change leads to an electrostatic change, whereby a positively charged amino acid becomes a more hydrophobic amino acid, which is hypothesized to disrupt filament formation. The C‐terminus of GFAP is thought to play a role in filament formation, but because the structure is currently unresolved, future studies will be necessary to better understand the role of the C‐terminus in protein function, and how variants located within this region might contribute to pathogenicity in Alexander disease. Many disease‐associated variants have been described in the C‐terminus of GFAP (Fig. 2B), as well as variants in paralogous proteins at the same or similar positions, highlighting the importance of the tail domain in Alexander disease and in other disease‐associated IF III proteins.

**Figure 4:**
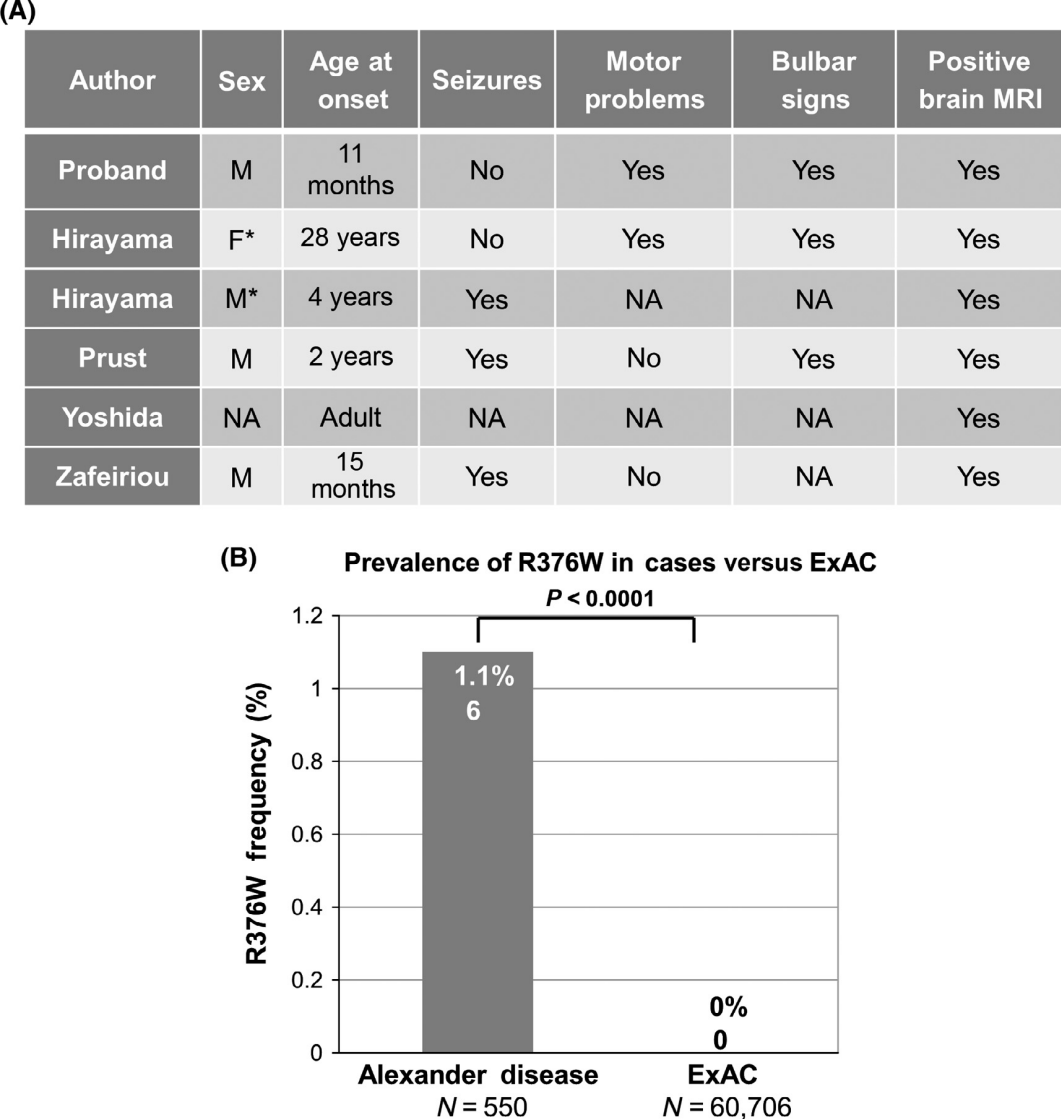
Spectrum and Prevalence of p.R376W Variants in Cases and Publically Available Databases. (A) R376W has been described in five previous Alexander disease cases. It can be observed that the phenotypes across these five cases are quite diverse, with differing age of onset and symptomology. * represents a mother and son who both harbor the p.R376W variant. NA is present when the information was not available. 34, 35, 36, 37 (B) p.R376W variants are overrepresented in Alexander disease (6/550) compared with the publically available Exome Aggregation Consortium (ExAC; 0/60,706; *P* < 0.0001) 22, suggesting overrepresentation in cases versus controls.

Interestingly, p.R376W has been reported in five individuals with variable onset Alexander disease in the scientific literature (Fig. 4A). The first case was a 33‐year‐old woman with progressive dysarthria and unsteady gait beginning at 28 years of age. She had subtle rhythmic ocular myoclonus, mild dysphonia, dysphagia, palatal myoclonus, ataxia and spastic gait, increased tendon reflexes, bilateral Babinski's signs, brain MRI showing high‐signal‐intensity lesions on T2‐weighted images in the cerebral white matter, brainstem, and hilus of the dentate nucleus, and a tumor with calcification in the left lateral ventricle around the foramen of Monro. The second case was her son, who presented with a convulsive seizure at 4 years of age. Brain MRI showed deep cerebral white matter abnormalities 34. The third case was a male who presented at 2 years of age with seizures, encephalopathy, bulbar symptoms, and typical brain MRI features for Alexander disease 35. The fourth case had clinically diagnosed adult‐onset Alexander disease; however, details pertaining to the case are not available 36. The fifth and final case was a 15‐month‐old male who presented with refractory status epilepticus. Until presentation, development was normal, and however, macrocephaly was noted. Brain MRI revealed diffuse bilateral and symmetric signal changes predominately of the frontal periventricular and subcortical white matter, and the basal ganglia, medulla, and cerebellar white matter 37. The two previously described infantile cases as well as the juvenile case presented with seizures. Our patient has not had any seizures to date, and although few cases have been described, he seems to have a less severe presentation in comparison with other infants with Alexander disease. While the vast majority of *GFAP* variants are associated with only one or two onset subtypes (infantile, juvenile, and adult), p.R376W has presented in all three onset subtypes. This could be a unique feature of p.R376W or may be due to the fact that many of the *GFAP* variants are rare and have not yet been seen in enough patients to see the same degree of clinical variation 2.

Although several Alexander disease cases have been described in the literature with the p.R376W variant, it has not been previously functionally validated and was reported by the genetic testing company as a VUS. These findings, along with the somewhat unusual presentation of the case described in this study, presenting during the infantile period with findings more representative of the juvenile group with abnormal brain MRI, psychomotor delay, and bulbar signs without signs of regression and signs of developmental progression, prompted functional validation. To confirm the disease‐causing nature of this variant and further investigate the mechanism of action, we used a stepwise approach of variant annotation with increasingly complex analyses. Using public data, we show that p.R376W has not been previously identified in the Exome Aggregation Consortium (ExAC; *n* = 60,706)^22^ emphasizing its rarity. Furthermore, there is a significant overrepresentation of p.R376W in cases (6/550; 1.1%) compared with the publically available database, ExAC (0/60,706; 0%; *P* < 0.0001; Fig. 4B). After cosegregation analysis with the parents of the child, it was determined that this variant was de novo*,* which is the typical mode of inheritance for Alexander disease; however, parental testing was important as there have been previous examples of parental transmission with adult‐onset Alexander disease 5. Using protein modeling techniques, we found the p.R376W variant in GFAP to be highly conserved across paralogs, with disease‐associated variants residing in similar locations in other paralogous proteins, suggesting the C‐terminal domain of GFAP is essential for protein function. Based on these findings, we proceeded with heterologous expression of GFAP in SW‐13 cells with immunofluorescence to examine the protein properties in vitro. These studies showed that p.R376W was no longer able to make fibers, rather it formed protein aggregates. These findings closely mimicked previously published Alexander disease variants including p.R239H 32, which has been previously modeled in mice, as well as p.S247P 5 and p.A253G 3, which have both been modeled in the same cellular model utilized in this study. In addition, p.S247P has been modeled in a homozygous and a heterozygous fashion in SW‐13 cells 5, both of which showed the same aggregate formation in vitro; and therefore, we believe that p. R376W works in a similar dominant negative fashion.

In summation, the rarity of this variant, the prevalence of p.R376W variant in cases of Alexander disease, the de novo presentation, the paralog analysis, and the cellular phenotype provide strong evidence that p.R376W is the cause of the abnormal brain MRI and phenotypes observed in our patient.

## Conclusions

A de novo GFAP missense variant was identified in a child presenting with hypotonia, developmental delay, and abnormal brain MRI. Paralog annotation, functional analysis, and frequency in Alexander disease cases versus publically available databases have provided substantial evidence that the p.R376W variant in GFAP is indeed responsible for our patient's phenotype. Due to the less severe presentation of Alexander disease in our patient, our experience supports the pursuit of genetic testing for Alexander disease when a suggestive brain MRI findings are present, but the phenotypic sequelae are less supportive of this diagnosis. In addition, following the new 2015 ACMG variant classification guidelines 38, the supporting functional evidence for p.R376W, described herein, should allow for the reclassification of p.R376W from a VUS to a pathogenic variant.

## Conflicts of Interest

The authors of this manuscript have no conflict of interests pertaining to the content of this manuscript.

## Supporting information


**Table S1.** GFAP Oligo list.
