# Supplementary material for: Functional characterization of a GFAP variant of uncertain significance in an Alexander disease case within the setting of an individualized medicine clinic
Source: Clin Case Rep. 2016 Aug 15;4(9):885–95. doi: 10.1002/ccr3.655 (PMC5018595; doi:10.1002/ccr3.655)
Supplement: Supplementary file 1 — Table S1. GFAP Oligo list. [file CCR3-4-885-s001.docx]

| Name of Oligo | Oligo Sequence |
| --- | --- |
| GIB-CAGS-GFAP-F1 | cattttggcaaagaattcctcgagcaggatggagaggagacgc |
| GIB-CAGS_GFAP-R1 | atttttggcagagggaaaaagatctcacatcacatccttgtgctcc |
| GIB-GFAP(A253G)-F1 | catgcatgaaggtgaagagtggtaccgctccaag |
| GIB-GFAP(A253G)-R1 | accactcttcaccttcatgcatgttgctgg |
| GIB-GFAP(R239H)-F2 | gagatccacacgcagtacgaggcaatggcgtccag |
| GIB-GFAP(R239H)-R2 | attgcctcgtactgcgtgtggatctctttcag |
| GIB-GFAP(R376W)-F1 | gggcgaggagaattggatcaccattcccgtg |
| GIB-GFAP(R376W)-R1 | gaatggtgatccaattctcctcgccctctagcag |
| GIB-GFAP(S247P)-F1 | atgaggccatggcgcccagcaacatgcatgaag |
| GIB-GFAP(S247P)-R1 | tgctgggcgccatggcctcatactgcgtgc |

**SUPPLMENTARY MATERIALS**

Supplementary Table 1. GFAP Oligo List
